# Supplementary figures and images for: Metabolite dynamics over the course of anti-tuberculosis treatment in individuals with mild and severe tuberculosis
Source: PLOS Glob Public Health. 2025 Oct 15;5(10):e0004925. doi: 10.1371/journal.pgph.0004925 (PMC12527214; doi:10.1371/journal.pgph.0004925)

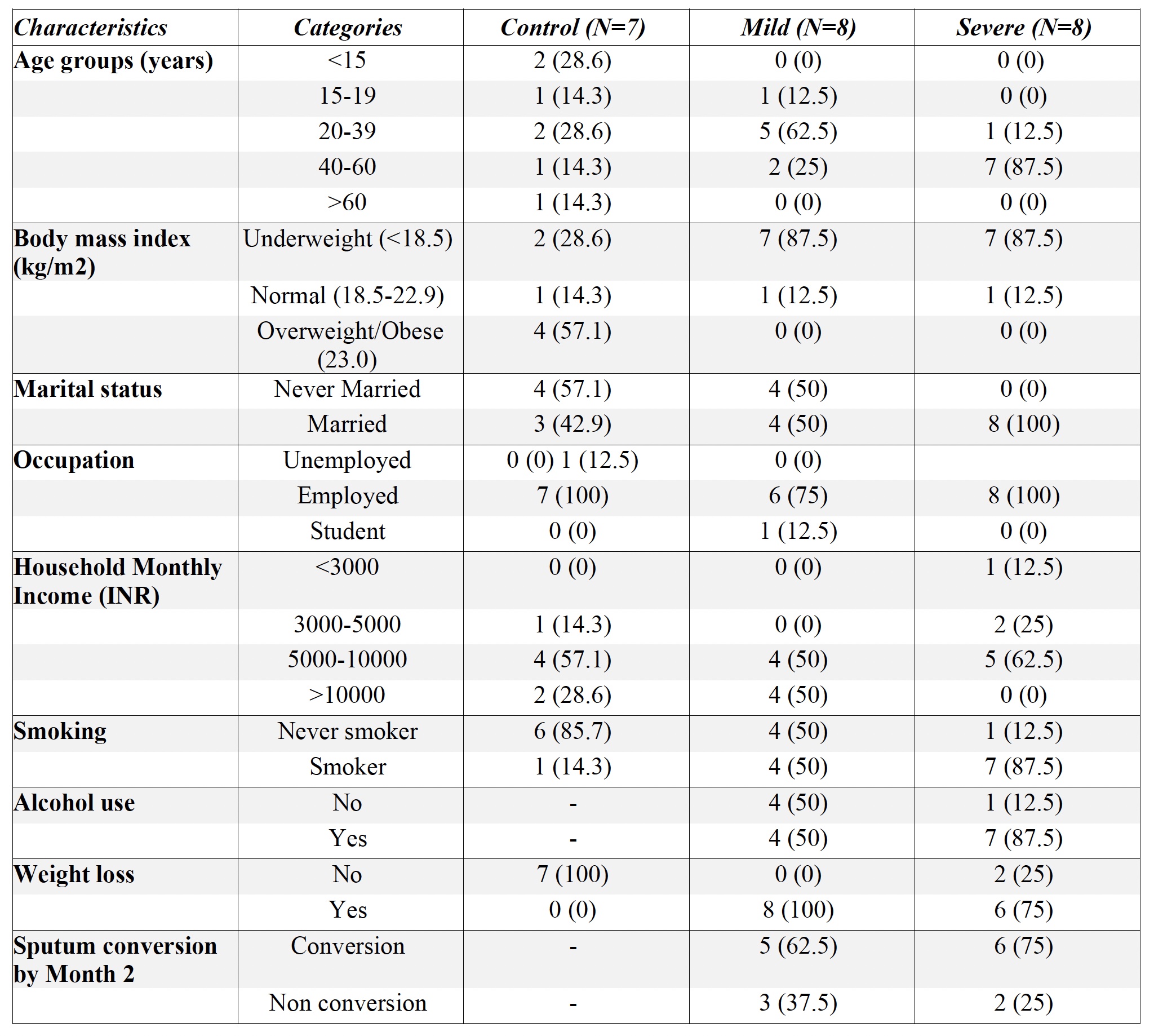

Supplement: S2 Table — A summary of the demographic characteristics of control, mild, and severe cases. Key variables include age, BMI, marital status, occupation, income, smoking, alcohol use, weight loss, and sputum conversion, highlighting differences in distributions across the groups. (JPG) [file pgph.0004925.s002.jpg]
