# Supplementary material for: Metabolite dynamics over the course of anti-tuberculosis treatment in individuals with mild and severe tuberculosis
Source: PLOS Glob Public Health. 2025 Oct 15;5(10):e0004925. doi: 10.1371/journal.pgph.0004925 (PMC12527214; doi:10.1371/journal.pgph.0004925)
Supplement: Attachment — Submitted filename: Response to Reviewers.docx [file pgph.0004925.s005.docx]

We would like to thank the two reviewers and the editor, Prof. Kiatichai Faksri, for their helpful and thoughtful comments on the previous version of this manuscript. Here, we provide responses to the points brought forward by the reviewers (in bold below) and highlight the revisions we have made to the manuscript. We have uploaded the updated manuscript text with and without track changes as advised.

1. Please provide separate figure files in .tif or .eps format.

**We have uploaded the figure files as .tif files to the correct dimensions, pixels and file sizes.**

2. Please review your reference list to ensure that it is complete and correct. If you have cited papers that have been retracted, please include the rationale for doing so in the manuscript text or remove these references and replace them with relevant current references. Any changes to the reference list should be mentioned in the rebuttal letter that accompanies your revised manuscript. If you need to cite a retracted article, indicate the article’s retracted status in the References list and also include a citation and full reference for the retraction notice.

**We have updated the reference list without any articles that have been retracted. Please refer to the track changed version to easily see the changes that were made.**

Additional Editor Comments (if provided):
Introduction: Although concise, the introduction would benefit from a brief contextualization of the global and national burden of tuberculosis, as well as a short explanation of the importance of metabolomics in understanding TB pathogenesis and treatment response. This will enhance the rationale for your study.

**We added more context in the introduction to highlight the global and national burden of tuberculosis along with a sentence highlighting the importance of metabolomics in understanding TB. These changes can be noted in the track changed version of the manuscript.**

Inclusion Criteria: Please clearly define the inclusion and exclusion criteria for study participants. For instance, were participants selected based on sex, age, or absence of comorbidities? Explicitly stating this will improve the reproducibility of your findings.

**The following was added to address the concern stated above: Participants in the study were categorized into three groups based on clinical and radiological severity of pulmonary tuberculosis (PTB) and infection status. *Group 1* (Severe group; N=8) comprised individuals with moderate to advanced chest X-ray (CXR) abnormalities and 3+ sputum smear positivity. *Group 2* (Mild group; N=8) included participants with mild CXR abnormalities and 2+/1+ sputum smear positivity. Both groups were randomly selected from eligible, newly diagnosed, smear-positive, culture-confirmed PTB patients, aged ≥18 years, with <7 doses of anti-TB treatment, and no history of TB or diabetes. Individuals who were HIV-positive, had contact with MDR-TB cases, were severely ill (Karnofsky score <40), or female were excluded. *Group 3* (Control group; N=7) consisted of IGRA-negative, healthy household contacts of PTB patients from Groups 1 and 2, who followed similar dietary patterns and served as metabolic controls. Controls were ≥18 years, had significant exposure to PTB cases (≥5 days/week or bed/room sharing), and were excluded if HIV-positive, symptomatic for TB in the past 3 months, had a history of diabetes, or were female.**

Results and Conclusion: The Results and Conclusion sections would benefit from additional explanation and clarity. Some of the findings are currently presented too briefly, which may hinder full understanding of their significance and implications.

**We added additional explanation and clarity that hopefully addresses the reviewer’s concerns. These changes can be found in the track changed version.**

In-Text Citations: Please review your in-text citations for accuracy. For example, the citation in line 118 currently refers to reference 27 but should be corrected to reference 2. A general review of all citation numbers is recommended to ensure consistency.

**All in-text citations were double checked to ensure accuracy and consistency. Changes can be found in the track changed version of the manuscript.**

Sample Size Justification: While you acknowledge the limited sample size, a brief justification or contextual note regarding sample size estimation—even within the scope of a pilot study—would strengthen the manuscript’s methodological transparency.

**The following was added to the methods and discussion to address the concern above from the reviewer: This study was designed as a pilot investigation utilizing retrospectively available samples, with the primary aim of exploring metabolic differences across varying severities of pulmonary tuberculosis (PTB).**

**The small sample size reflects the strict application of inclusion and exclusion criteria, which were necessary to ensure well-defined and homogenous study groups while minimizing potential confounders such as comorbidities (e.g., diabetes, HIV). Also, due to the retrospective nature of sample availability, only a limited number of eligible participants met the stringent criteria. Despite the small size, this focused and carefully selected cohort allows for meaningful exploratory analysis, and we are optimistic that the findings will help inform the design and power calculations of larger, prospective studies.**
